# Characteristics and outcome of infants born to mothers with SARS-CoV-2 infection during the first three waves of COVID-19 pandemic in northern Iran: A prospective cross-sectional study

**DOI:** 10.1016/j.amsu.2022.103839

**Published:** 2022-05-23

**Authors:** Roya Farhadi, Vajiheh Ghaffari, Shahrokh Mehrpisheh, Mahmood Moosazadeh, Mohammadreza Haghshenas, Aghdas Ebadi

**Affiliations:** a Pediatric Infectious Diseases Research Center, Communicable Diseases Institute, Mazandaran University of Medical Sciences, Sari, Iran; b Health Sciences Research Center, Mazandaran University of Medical Sciences, Sari, Iran; c Antimicrobial Resistance Research Center, Communicable Diseases Institute, Mazandaran University of Medical Sciences, Sari, Iran; d Department of Obstetrics and Gynecology, Mazandaran University of Medical Sciences, Sari, Iran

**Keywords:** COVID-19, Newborn, Outcome assessment, Sars-cov-2, Viral load, SARS-CoV-2, Severe Acute Respiratory Syndrome Corona-Virus 2, COVID-19, Coronavirus Disease 2019, RT-PCR, Reverse Transcription – Polymerase Chain Reaction, NICU, Neonatal Intensive Care Unit, ICU, Intensive Care Unit

## Abstract

**Background:**

Despite the rapid increase in knowledge about coronavirus disease 2019 (COVID-19), there is limited data on vertical transmission, viral loads in mother-neonate pairs, and health outcomes. We aimed to describe the characteristics, viral loads, and short-and mid-term outcomes of neonates born to mothers with confirmed COVID-19 infection in northern Iran.

**Materials and methods:**

In a cross-sectional study, we prospectively collected and analyzed the clinical features, reverse transcription-polymerase chain reaction (RT-PCR) results, viral loads, and outcomes of 60 neonates delivered by 58 SARS-CoV-2 infected pregnant women in maternity hospitals of Mazandaran University of Medical Sciences (northern Iran) during first three waves of the pandemic from March 1 to December 31, 2020. We assessed neonates' short and mid-term outcomes up to 24 months after the pandemic. We also described the timing of mother-to-infant transmission based on the classification presented by the World Health Organization.

**Results:**

Of the 17767 deliveries, 58 mothers had confirmed and probable COVID-19 infection. Twenty (33.3%) neonates were positive for SARS-CoV-2, two of whom had possible in utero transmission. Twenty-five (41.2%) neonates were preterm, most of whom were born during the first and second waves in which mothers were critically ill. 19 (31.7%) patients needed resuscitation in the delivery room. 34 (56.7%) neonates were isolated in the neonatal intensive care unit. We observed a significant relationship between the maternal and neonatal viral load (correlation coefficient = 0.983, P = 0.00). No neonatal death was observed and all babies had a good outcome.

**Conclusions:**

The results showed that vertical transmission of SARS-CoV-2 is possible but rare. Regional factors and severity of mother's disease may influence the clinical course of neonates. With increasing experience, proper observance of health precautions, and rapid development of evidence-based response systems for regional and global disasters, the transmission rate of SARS-CoV-2 from mother to newborn is reduced.

## Highlights

• Data describing the effect of SARS-CoV-2 in newborns are scarce.
• The mid-term and long-term outcomes of neonates exposed to SARS-CoV-2 infection are still unknown.
• We described characteristics and outcomes of SARS-CoV-2 infection in neonates who were born to infected mothers.
• Vertical transmission is rare but possible.
• Neonatal short-term and mid-term outcome is good.

## 1 Introduction

Human infection from (Severe Acute Respiratory Syndrome Corona-Virus 2, SARS-CoV-2) was first reported in China. On February 11, 2020, World Health Organization (WHO) announced that the novel coronavirus would be called COVID-19 [[1], [2], [3]]. On February 19, Iranian officials announced the COVID-19 outbreak in the country [3,4].

Mazandaran is one of the most densely populated provinces in the north of Iran. It covers an area of 23,842 km^2^ and has been also identified as one of the most affected regions since the announcement of the virus's spread [4].

After the first wave of infection, Iran became one of the first countries in the world to report a second wave of coronavirus in June 2020 and faced the third wave of the outbreak in the first days of October [5,6].

To date, with the current evidence-based predominantly on case series, there is little knowledge about perinatal and neonatal COVID-19 infection and its short-term and long-term harm to offspring. Therefore, it is highly essential to identify clinical symptoms and outcomes of COVID-19 infection in neonates born to affected mothers and neonatologists need more clinical, and virological data to manage COVID-19 [[7], [8], [9], [10], [11], [12]]. On February 8, 2021, WHO proposed a consensus classification system for the timing of infection occurrence (in utero, intrapartum, and early postnatal) which is classified as follows: confirmed, possible, unlikely, and indeterminate [13].

On March 7, 2020, the first pregnant mother with severe COVID-19 infection was admitted to Mazandaran province, Iran. The pregnancy was terminated, and a preterm newborn was born. Unfortunately, the mother died due to respiratory failure. Neonate's nasopharyngeal swab RT-PCR test was positive for SARS-CoV-2 24 h after birth and this case raises concerns about the possible vertical transmission of COVID-19 [14,15]. Then, through the university, the necessary instructions were sent to all hospitals in the province according to the national protocol for approaching neonates born to mothers with COVID-19 infection. Babies were also registered in the national registry system.

The purpose of this study is to provide a characterization of neonates born to mothers with COVID-19 infection during the first three waves of disease in northern Iran focusing on clinical presentation, the possibility of vertical transmission, and outcomes after 24 months of follow up.

## 2 Materials and Methods

We conducted a prospective cross-sectional study on neonates born to mothers with confirmed or probable COVID-19 infection based on WHO case definitions [16]. We included all the above mothers and newborns who were admitted to hospitals in Mazandaran in northern Iran from March 1 to December 31, 2020, by census method and followed the infants until March 1, 2022. This study was approved by the ethics committee of research of Mazandaran University of Medical Sciences (IR.MAZUMS.REC.1399.7375). Written informed consent was obtained from neonates’ parents. This study is compliant with the STROCSS criteria [17].

The management of newborns was under updated protocols provided by the national mother and newborn health committee. Data regarding epidemiologic and clinical features were obtained from the neonatal COVID-19 registration system of Mazandaran University of Medical Sciences. Neonatal nasal and throat swab samples were tested for SARS-CoV-2. Cases in which amniotic fluid or umbilical cord were received for RT-PCR testing were also recorded.

All neonates born to infected mothers were isolated and two nasopharyngeal samples were taken from the neonates during the first and second day of life, respectively. If the test was positive, the test was repeated every 48 h. When the baby was in good condition and had two negative RT-PCR tests, the baby would be discharged with the necessary recommendations.

Patients’ data included time of admission (first, second, and third wave), gestational age, sex, birth weight, Apgar score, mode of delivery, symptoms or diagnosis at onset, need for ventilator support, feeding status, clinical course, and duration of hospital stay. The percentage of neonates who had positive nasopharyngeal RT-PCR results was reported based on the WHO classification [13]. Outcomes at the time of data closure include survival (maternal and neonatal), retinopathy of prematurity, hearing screening results, and any medical problems for patients in whom data were complete according to follow-up in clinical or virtual visiting.

### 2.1 Statistical analysis

A descriptive analysis was done using SPSS version 25(IBM Corp., Armonk, N.Y., USA). Chi-squared test and Fisher's exact test were used to compare the categorical variables. Quantitative variables between the two groups were compared using the Mann-Whitney *U* test, and the Kruskal-Wallis test was used to compare quantitative variables in more than two groups. Spearman correlation coefficient was used to assess the relationship between maternal and neonatal viral load. A P-value of less than 0.05 was considered significant.

## 3 Results

**Fig. 1:**
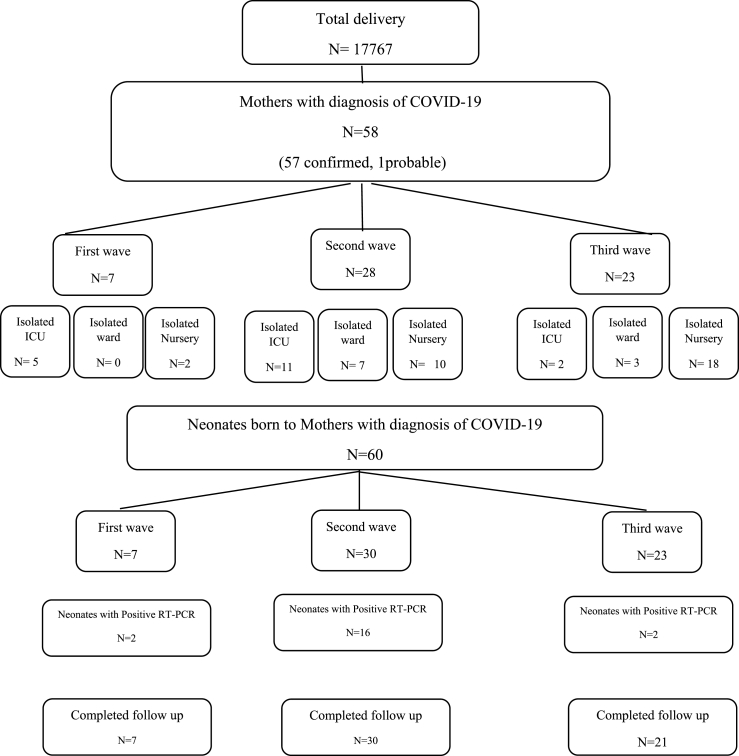
Study profile of positive mothers with COVID-19 and their newborns.

Of total of17767 deliveries during the study period, there were 58 (0.3%) mothers were admitted with a probable or confirmed diagnosis of COVID-19. The mean maternal age (±SD) and mean gestation (±SD) were 27.52 ± 4.67 years and 36.40 ± 2.70 weeks respectively. Forty-one mothers had a history of contact with COVID-19 patients. Duration of mothers’ illness before delivery was 5.12 ± 3.17 (range 1–14) days. Cesarean delivery was performed in 65.5% of mothers. The Amniotic fluid RT-PCR test was positive in 4 mothers. Thirty-one percent of mothers were admitted to the ICU and 17.3% to the isolation ward. In 51.7% of cases, the mothers were transferred to the nursery with their neonates. Two mothers gave birth to twins and a total of 60 newborns were born. During the study period, unfortunately, one case of maternal death was reported. The profiles of hospitalized mothers and neonates are shown in Fig. 1.

### 3.1 Characteristics and clinical manifestations of neonates born to mothers with COVID-19

A total of 60 neonates were born to mothers with SARS-CoV-2 and two pairs were twins. Twenty–five babies (41.7%) were born preterm. The mean weight of neonates was 2782.92 ± 746.77 g. Skin-to-skin contact was performed for 35 (58.3%) newborns during the second and third waves of the disease. Thirty-seven neonates (61.7%) were male. Nineteen (31.7%) babies needed resuscitation at birth. The median Apgar score was 8(range 7–10) and 9(range 8–10) in 1 and 5 min after birth respectively. Thirty-four (56.7%) neonates were isolated in the NICU and 7 (11.7%) in the neonatal ward. Nineteen (31.7%) newborns were collocated with their mothers in the rooming-in ward. Table 1 shows the characteristics of infected mothers and newborns during three waves of the disease separately.

**Table 1:** Characteristics of infected mothers with COVID-19 and the newborns.

Variable	first wave	second wave	third wave	P-value
Mothers ‘age(year) Mean ± SD CI 95%	30.29 ± 4.92 25.73–34.84	26.63 ± 4.31 25.02–28.25	27.83 ± 4.88 25.71–29.94	0.16
Mothers’ symptom duration (day) Mean ± SD CI 95%	7.86 ± 4.67 3.54–12.18	5.17 ± 2.99 4.05–6.29	4.22 ± 2.44 3.16–5.28	0.13
Contact history	N = 5(71.4%)	N = 17(60.7%)	N = 19(82.6%)	0.13
Positive RT-PCR mothers	N = 6(85.7%)	N = 28(100%)	N = 23(100%)	0.26
Mode of delivery (C/S)	N = 5(71.4%)	N = 17(60.71%)	N = 16(69.6%)	0.56
Gestational age (weeks) Mean ± SD CI 95%	32.71 ± 2.92 30.01–35.42	36.47 ± 2.36 35.59–37.35	37.43 ± 2.10 36.52–38.35	0.002
Admission in ICU	N = 5(71.4%)	N = 11(39.2%)	N = 2(8.7%)	0.006
Positive RT-PCR of Amniotic fluid	N = 3(42.9%)	N = 1(3.5%)	N = 0	0.004
Neonates				
SSC	N = 2(28.6%)	N = 16(53.3%)	N = 17(73.9%)	0.07
Sex (male)	N = 2(28.6%)	N = 20(66.7%)	N = 15(65.2%)	0.15
Weight Mean ± SD (gram) CI 95%	2094.29 ± 672.18 1472.62–2715.95	2882.17 ± 810.01 2579.70–3184.63	2863.04 ± 580.03 2612.22–3113.87	0.04
Apgar (1 min) Mean ± SD range	8.43 ± 0.78 7–9	8.63 ± 0.71 7–10	8.61 ± 0.65 7–9	0.76
Apgar score(5 min) Mean ± SD range	9.86 ± 0.37 9–10	9.93 ± 0.25 9–10	9.83 ± 0.49 8–10	0.67
Need to resuscitation	N = 5(71.4%)	N = 9(30%)	N = 5(21.7%)	0.05
Isolation in NICU Ward nursery	N = 6(85.7%) N = 0 N = 1(14.3%)	N = 23(76.7%) N = 4(13.3%) N = 3(10%)	N = 5(21.7%) N = 3(13%) N = 15(65.2%)	0.00
Hospital stay (day) Mean ± SD CI 95%	21.29 ± 12.89 9.36–33.21	6.37 ± 4.16 5.18–8.29	3.91 ± 2.96 2.63–5.20	0.00
Positive RT-PCR	N = 2(28.6%)	N = 16(53.3%)	N = 2(8.7%)	0.003
Feeding status EBF PBF FF	N = 2(28.6%) N = 3(42.9%) N = 2(28.6%)	N = 19(63.3%) N = 9(30%) N = 2(6.7%)	N = 17 (73.9%) N = 6(26.1%) N = 0	0.06
Prematurity	N = 6 (85.7%)	N = 14(46.7%)	N = 5(21.7%)	0.008
Mother death	N = 1 (14.3%)	0	0	0.11

Abbreviations: RT-PCR= Reverse Transcription – Polymerase Chain Reaction, C/S=Caesarean section, ICU=Intensive Care Unit, SSC=Skin to Skin Contact, NICU= Neonatal Intensive Care Unit, EBF = Exclusive Breast Feeding, PBF=Partial Breast Feeding, FF=Formula Feeding.

In total, 20(33.3%) neonates had positive nasopharyngeal COVID-19 RT-PCR test and two of whom were related to the first 24 h and considering that the mothers’ amniotic fluid was also positive, there was a very high suspicion of vertical transmission for them and these two babies were classified as possible in utero transmission. The first baby was born in the first days of the epidemic in Iran. Mother was very ill and died. The second case was born to a mother who had mild symptoms but showed a high viral load on the nasopharyngeal and amniotic fluid RT-PCR test during the second wave of the epidemic. This baby had a positive nasopharyngeal RT-PCR test on the first day of life.

Results of the amniotic fluid RT-PCR test were also positive in two other mothers during the first wave of the disease. Both mothers were relatively ill and had preterm deliveries and the infants were admitted to the NICU, but all the neonatal RT-PCR results were negative. Unfortunately, at that time we had no access to serologic kits for the detection of COVID-19 antibodies. These two babies were considered unlikely in utero transmission. One neonate who was born to a mother with a negative amniotic fluid RT-PCR test had a positive nasopharyngeal RT-PCR test at age 16 h but the second sample at 36 h of life was negative so she was considered unlikely in utero transmission. In 16 neonates the nasopharyngeal RT-PCR test was performed on the second or third day of life and in one newborn, it was possible to perform the test at 7 days of age, which all had positive results. Of them, 9 neonates were classified in indeterminate in utero/unlikely intrapartum transmission category, 7 neonates in unlikely early post-natal transmission category, and one baby in indeterminate early post-natal transmission category.

None of the neonates received any antiviral therapy. Of the total number of infants born to infected mothers, 19 had no medical problems.

Neonates with positive RT-PCR tests were admitted with diagnoses including pneumomediastinum, seizure, RDS, neonatal abstinence syndrome, sepsis, TTN, and jaundice, and three of them were discharged home with mothers. No neonatal death was observed during the study period and all neonates were discharged in good general condition. In terms of nutritional status, 38 (63.3%) neonates were exclusively breastfed, 4 (6.7%) were formula-fed and 18(30%) were partially breastfed. The mean hospital stay for neonates was 7.35 ± 7.51 (average 2–40) days. Except for two infants who could not be reached access to their families, all infants were followed up by telephone and visits to the clinic after discharge. Table 2 shows individualized details of COVID-19 positive neonates born to infected mothers.

**Table 2:** Individualized details of COVID-19 positive neonates born to infected mothers.

Details/neonates	1	2	3	4	5	6	7	8	9	10	11	12	13	14	15	16	17	18	19	20
wave	1	1	2	2	2	2	2	2	2	2	2	2	2	2	2	2	2	2	3	3
sex	female	female	male	Male	male	male	male	Male	male	Female	male	male	male	male	male	male	male	female	female	male
Gestation(weeks)	32	35	39	38	34	38	38	38	39	39	39	39	36	33	38	36	36	36	38	34
Birthweight (g)	2350	2420	3890	3400	2000	3600	4000	3300	3730	3915	3200	3700	3200	2800	3470	2500	2200	2880	2450	2130
Mode of delivery	C/S	C/S	C/S	C/S	NVD	NVD	NVD	NVD	NVD	NVD	C/S	NVD	C/S	C/S	NVD	NVD	NVD	C/S	C/S	C/S
Apgar at 1–5 min	8–9	9–10	9–10	9–10	9–10	9–10	8–10	8–10	9–10	9–10	10–10	9–10	9–10	8–9	9–10	9–10	8–10	9–10	9–10	9–10
Duration of mothers symptom before delivery (day)	10	8	2	3	4	2	4	4	7	14	3	4	4	2	3	2	3	5	8	10
Amniotic fluid RT-PCR	positive	negative											positive							
Day of baby RT-PCR positivity	1,4,7,14	1	2	7	3	3	3	2	3	3	2	2	1,3,7,10	3	2	2	2	2	2	3
Place of isolation	NICU	NICU	NICU	NICU	NICU	NICU	NICU	NICU	NICU	NICU	ward	RI	NICU	NICU	ward	NICU	RI	ward	NICU	NICU
Breastfeeding status	FF	PBF	EBF	EBF	EBF	EBF	EBF	EBF	EBF	EBF	PBF	EBF	PBF	PBF	EBF	FF	EBF	EBF	EBF	EBF
Need to ventilation	CPAP	CPAP																		
Clinical diagnosis	RDS+ PM	RDS	Seizure	Sepsis	TTN	seizure	TTN	TTN	Sepsis	TTN			PM	RDS	sepsis	NAS		Icter	sepsis	TTN
Maternal cycle threshold			27	15	27	24	15	15	29	37	32	32	29	27	38	27		38		15
Neonatal cycle threshold			24	14	24	24	14	14	29	32	32	29	29	27	37	27		37		14
Duration of hospital stay	30	13	8	10	5	8	4	3	9	8	4	3	14	13	12	3	2	3	7	5
Need to resuscitation	yes	yes	No	No	yes	no	no	No	no	no	no	no	no	yes	no	no	no	no	no	yes
Neonatal outcome	good	good	good	Good	good	good	good	Good	good	good	good	good	good	good	good	N/F	good	good	good	good
Maternal outcome	Died	good	good	Good	good	good	good	Good	good	good	good	good	good	good	good	good	good	good	good	good
Mother's admission place	ICU	ICU	RI	RI	ICU	ward	ward	RI	RI	RI	ward	RI	ward	ward	RI	ward	RI	RI	RI	ICU
Timing of transmission	Possible IU	Unlikely IU	Indeterminate IU/Unlikely IP	Indeterminate EPN	Unlikely EPN	Unlikely EPN	Unlikely EPN	Indeterminate IU/Unlikely IP	Unlikely EPN	Unlikely EPN	Indeterminate IU/Unlikely IP	Indeterminate IU/Unlikely IP	Possible IU	Unlikely EPN	Indeterminate IU/Unlikely IP	Indeterminate IU/Unlikely IP	Indeterminate IU/Unlikely IP	Indeterminate IU/Unlikely IP	Indeterminate IU/Unlikely IP	Unlikely EPN

Description of data.

Abbreviation: C/S= Caesarean section, NVD=Normal Vaginal Delivery, NICU= Neonatal Intensive Care Unit, RI = Rooming in with mother, EBF = Exclusive Breast Feeding, PBF=Partial Breast Feeding, FF=Formula Feeding, CPAP=Continuous Positive Airway Pressure, RDS = Respiratory Distress syndrome, TTN = Transient Tachypnea of Newborn, NAS= Neonatal Abstinence Syndrome, IU=In Utero, IP=Intrapartum, EPN = Early Postnatal.

### 3.2 Outcome assessment

All the babies in the follow-up were in good general condition and had no problem up to March 1, 2022. Three infants who had a negative RT-PCR test during the first wave of the disease, were infected and hospitalized in September 2021 during the outbreak of the Delta variant of COVID-19 in Iran and were discharged in good general condition. An infant from the RT-PCR-positive neonatal group also developed COVID-19 infection during this period but recovered with outpatient treatment. The result of the otoacoustic emission (OAE) hearing screening test in one neonate was abnormal but the repeated OAE test at three months was normal. Except for one infant who showed retinopathy of prematurity, the rest of the preterm infants had a normal retinal exam.

IgM and IgG antibodies were checked during admission for only a small number of neonates who had positive RT-PCR tests, but no serological test was positive.

Viral load was recorded in 16pairs of mothers and neonates who had positive RT-PCR tests. The results showed a mean (±SD) of 26.69 ± 8.09 in the mothers and 25.44 ± 7.85 in the neonates that had a significant relationship (correlation coefficient = 0.983, P = 0.00).

## 4 Discussion

In this study, we present the clinical characteristics and outcomes of neonates born to women with COVID-19 infection in a province with a high incidence of maternal SARS-CoV-2.To the best of our knowledge, this is the first and large Iranian cohort of newborns born to mothers with confirmed COVID-19 infection which classified the timing of transmission based on WHO definition and followed up neonates about 24 months after the pandemic. SARS-CoV-2 was detected by nasopharyngeal swab samples in 33.3% of newborns, and two of them were high probable of vertical transmission. Moreover, we report no adverse outcomes in the follow-up of these neonates. Overall, hospitals in the province tested only symptomatic pregnant women for SARS-CoV-2 and we could not able to universal screen all pregnant mothers admitted for delivery. During the first wave of the pandemic, the mothers were critically ill and admission to ICU was more than two other waves. In the second wave, more symptomatic mothers were screened and more neonates were affected. Through the third wave despite an extension of screening, mothers presented with mild symptoms, and affected newborns were less than two previous waves.

In a cohort study in the US, 120 neonates born to mothers who tested positive for SARS-CoV-2 were studied. None of the neonates were positive for COVID-19 and only 17% were preterm [18]. They could screen all mothers who were admitted for delivery while we only screened symptomatic mothers. A study in India on 65 neonates born to COVID-19 positive mothers showed that 10.7% of neonates had positive COVID-19 RT-PCR test [19]. Chen et al. reported clinical records of nine pregnant women with COVID-19 pneumonia in China. All patients had a cesarean section and no neonatal death was reported. The presence of SARS-CoV-2 had tested in 6 neonates that none of them was positive [20]. Another case study from China described the clinical course of four infants born to pregnant women with COVID-19 infection and showed that all were term and no positive RT-PCR test for COVID-19 was reported [21]. Yu et al. in a retrospective study in China reported the neonatal outcome of 7 pregnant women with COVID-19 and only one neonate was infected with SARS-CoV-2 [22]. In another cross-sectional study in the US, of 118 mothers 38% were positive for SARS-CoV-2 and only 6.7% of newborns had tested positive for SARS-CoV-2, and 73% of neonates roomed in with their mothers [23]. A prospective national cohort study of babies with confirmed COVID-19 infection in the UK, identified that infection in the first 7 days after birth was not common and they identified only two babies with possible vertical transmission. They resulted that the severity of SARS-CoV-2 infection in neonates might have been related to other conditions like prematurity or ethnicity. In their study neonates from black, Asian, and mixed ethnic groups account for half of the neonates admitted with COVID-19 infection [24,25]. These results also may be accountable for the results of our study. On the other side, intrapartum or early post-natal occurrence of infection in our study may be due to viral contamination in the delivery room, exposure to infected mothers or caregivers especially in settings where the spaces for baby care and isolation are limited.

In our study, approximately 42% of newborns were born prematurely which is higher than the UK and US studies. Prematurity may be secondary to an obstetric decision due to the severity of the infection in the mother (as we reported during the first wave in our region).In mildly symptomatic mothers (what we observed in the third wave) it may not increase the risk of delivering prematurely [10,26]. Results of a large cohort analysis of 101 neonates born to mothers positive for SARS-CoV-2 infection in New York showed that the small number of mothers with severe COVID-19 transfer SARS-CoV-2 to their newborns that are in contrast with our study [27]. A previous report showed that the severity of the mother's COVID-19 infection may increase the concern for vertical transmission [28]. The role of the duration of a mother's infection before delivery on the rate of positive test results in neonates is also unclear [29,30]. In addition, the viral load may be another important factor in the transmission of infection [31,32]. A study showed that the maternal viral load is not associated with the positivity status or severity of the disease in neonates [19]. We showed that the cycle threshold and the number of viral loads had a significant relationship between mothers and affected neonates.

We reported two babies with possible in utero transmission. A meta-analysis reported the average pooled incidence of vertical transmission of 16 per1000 newborns. They stated although the rate of vertical transmission is low, the overall incidence could be higher than what they estimated [33].

Unfortunately, in the first and second waves of the pandemic due to resource limitations like not access to a milk bank in the region and lack of experience we had to isolate all newborns, and the rate of skin-to-skin contact and breastfeeding was low in our study. The breastfeeding rate increased in our region with the use of mobile-assisted virtual bonding [34], increasing experience during the third wave, and early detection of symptomatic mothers.

Neonates were admitted with different diagnoses in our research, but it is difficult to differentiate COVID-19 symptoms from symptoms of RDS, TTN, or sepsis [28]. Two of our neonates with positive PCR tests presented with seizures. Vivanti et al. reported transplacental transmission of SARS-CoV-2 from a mother to the fetus and the neonate presented with neurological manifestation [35].

All these side effects of this pandemic had a positive effect on our NICU like the rapid development of virtual communication for parents. There is a need for supporting a neonatal international collaborative group to promote the exchange of information for informed local implementation of all aspects of neonatal cases for regional disasters [36].

### 4.1 Strength and limitations of the study

The strength of this research includes a relatively large sample size as an Iranian experience, a longer time of outcome assessment, and an assessment of the possibility of subsequent COVID-19 infection.

The limitation of our research was the limitation of resources for screening all pregnant mothers. The cities that were far from the center of the province did not have access to the kit for testing in the first 24 h. We were also unable to perform rectal swabs, cerebrospinal fluid, or other sterile samples of RT-PCR in neonates.

## 5 Conclusion

Possible vertical transmission of SARS-CoV-2 is rare and the neonatal outcome is good. With proper education of health care workers and families, the risk of transmission is reduced. Social, economic, and regional factors must be incorporated into policy decisions and more evidence is needed to assess the risk of vertical and horizontal transmission of SARS-CoV-2.

## Availability of data and materials

All data collected during this study are available from the corresponding author upon reasonable request.

## Ethical approval

Ethical approval was obtained for this report from the research ethics committee of Mazandaran University of Medical Sciences (IR.MAZUMS.REC.1399.7375).

## Sources of funding

Not applicable.

## Author contribution

Roya Farhadi collected the newborn's data, organized the research ethics approval, and developed the first draft manuscript. Vajiheh Ghaffari and Shahrokh Mehrpisheh provided the data regarding the follow-up. Mahmood Moosazadeh analyzed and interpreted the patients' data. Mohammadreza Haghshenas performed the virological examinations. Aghdas Ebadi was responsible for mothers' data collection. All authors agreed on the final submitted version of the study.

## Consent

The approval of the Ethics Committee for Research was obtained from Mazandaran University of Medical Sciences. (No. 1399.7375). Informed consent was obtained from patients’ guardians.

## Registration of research studies

Name of the registry: ethics committee of Mazandaran University of Medical Sciences.

Unique Identifying number or registration ID: IR.MAZUMS.REC.1399.7375.

1. Hyperlink to your specific registration (must be publicly accessible and will be checked): Not applicable

## Guarantor

Dr. Roya Farhadi.

## Provenance and peer review

Not commissioned, externally peer-reviewed.

## Declaration of competing interest

The authors declare that they have no competing interests.
